# A cheap and non-destructive approach to increase coverage/loading of hydrophilic hydroxide on hydrophobic carbon for lightweight and high-performance supercapacitors

**DOI:** 10.1038/srep18108

**Published:** 2015-12-08

**Authors:** Liuyang Zhang, Hao Gong

**Affiliations:** 1 Department of Materials Science and Engineering, National University of Singapore, Singapore 117576, Singapore

## Abstract

Carbon-based substrates offer unprecedented advantages in lightweight supercapacitors. However, it is still challenging to achieve high coverage or loading. Different from the traditional belief that a lack of defects or functional groups is the cause of poor growth on carbon-based substrates, we reckon that the major cause is the discrepancy between the hydrophilic nature of the metal oxide/hydroxide and the hydrophobic nature of carbon. To solve this incompatibility, we introduced ethanol into the precursor solution. The method to synthesize nickel copper hydroxide on carbon fiber paper employs only water and ethanol, in addition to nickel acetate and copper acetate. The results revealed good growth and tight adhesion of active materials on carbon fiber paper substrates. The specific capacitance and energy density per total weight of the active material plus substrate (carbon fiber paper, current collector) reached 770 F g^−1^ and 33 Wh kg^−1^ (1798 F g^−1^ and 54 Wh kg^−1^ per weight of the active materials), owing to the high loading of active material and the light weight of carbon fiber paper. These results signified the achievability of light, cheap and high-performance supercapacitors by an environmental-friendly approach.

Lightweight and low-cost energy storage devices have gained wide interest as they are important in a variety of applications ranging from portable consumer electronics to vehicles. Supercapacitors have been the paradigm energy storage devices because of their desirable properties including high power density, fast charge-discharge rate and excellent safety^1^,^2^,^3^,^4^,^5^,^6^. To further enhance the overall performance of supercapacitors, a suitable match of both superior electrode materials and current collectors should be addressed.

Pseudocapacitive transition metal oxide/hydroxide electrode materials, which can offer high specific capacitance and high energy density, have been explored multitudinously. Binary metal compounds (oxides^7^, hydroxides^8^, and sulfides^9^) with multiple oxidation states have been reported to display much better electrochemical performance than single metal components^10^. Our group found that nickel copper oxide/hydroxide could exhibit better performance than nickel oxide/hydroxide^11^,^12^.

For current collectors (substrates), using lightweight conductors is advantageous because it decreases the total weight of the device. This is important because the energy storage per total weight of an entire device (including substrate) is the main concern of industry and users. Currently, metal foams (such as nickel foam) are generally employed to grow electrode materials. The remarkable advantage of using a metal foam substrate is that electrode materials can be grown on the substrate with high loading and excellent coverage. However, metal foam substrates have high mass density. For example, the high mass density of nickel foam (262 mg cm^−3^ for commercial foam with a thickness of around 1.6 mm) leads to a low gravimetric capacitance of 12–200 F g^−1^ per total weight of the electrode and substrate despite the high specific capacitances of the active electrode materials. This value (12–200 F g^−1^) is calculated based on the results published (including ours). Therefore, it is desirable to grow electrode materials on lightweight current collectors (substrates).

Carbon fiber papers (CFPs), which consist of a network of microsized carbon fibers, have been extensively employed in proton-exchange-membrane fuel cells, sensors and field-effect devices^13^,^14^. Their unique interconnected porous structures allow them to immobilize active materials for the charge transfer. Their fascinating properties such as low sheet resistance (0.26 Ω cm^−2^)^15^, large surface area, good physical strength, biocompatibility and lightweight architecture^16^ enable them to be employed as versatile scaffolds/current collectors for supercapacitor applications. Additionally, to overcome the low intrinsic conductivity of metal oxide/hydroxide, direct growth of such electrode materials on conductive substrates is needed. CFP, with reasonably high conductivity, is an excellent candidate for use as a light-weight current collector as it provides porous channels for low-resistant ion diffusion and nano-sized skeletons for fast electron transfer.

However, the robust growth of electrode materials on CFP has always been a great challenge due to the surface incompatibility between the carbon fibers and the electrode materials^17^. To improve their surface compatibility, oxidative treatments of carbon materials (such as using acids) have been attempted previously. Oxidative treatments could introduce numerous structural defects and oxygen-containing groups on the surfaces of carbon materials, facilitating the growth of metal oxide/hydroxide^18^. However, attaining complete coverage of electrode materials on carbon substrates remains challenging in practice because of the lack of affinity between electrode materials and carbon/graphene. Besides, whether stronger interactions between the two materials guarantee superior cycling performance is still unknown.

Based on the fact that CFP is hydrophobic while metal hydroxide is hydrophilic, we wonder if excellent growth of metal hydroxide on carbon fiber paper can be achieved by using a suitable solvent that wets both materials well. Ethanol has a polarity smaller than water, so its use may alleviate the difference. Thus, experiments were designed to explore the effect of ethanol on the growth of material on CFPs.

Also, during the synthesis of metal hydroxide, in addition to metal precursor(s), other chemicals are also used typically. One type of such chemicals used serves the role of a precipitant. Examples of precipitants used include urea, hexamethylenetetramine (HMT), ammonia, NaOH, glycine, n-butyl amine, L-lysine and L-arginine^19^,^20^,^21^. However, these precipitants are usually expensive and needed to be removed by further annealing and additional washing. Another chemical used is the solvent. It has been reported that solvents are crucial in synthesizing metal oxide/hydroxide, and organic solvents are generally employed^22^,^23^,^24^. It will be advantageous from an environmental and economic perspective if the use of chemicals like precipitants and unsafe organic solvents can be avoided during synthesis. This is especially important for industrial mass production.

In this work, we used only water, in addition to metal precursors (nickel acetate and copper acetate), for the hydrothermal synthesis of nickel copper hydroxide. To solve the problem of poor growth or coverage of nickel copper hydroxide on CFP, ethanol was introduced. The effect of ethanol on the formation, size and morphology of nickel copper hydroxide has been studied. We found that the introduction of ethanol led to uniform growth, high capacitance and high energy density. Remarkably, very high specific capacitance (770 F g^−1^) and energy density (33 Wh kg^−1^) per weight of the whole device (excluding electrolyte and packaging) are obtained, owing to the high loading (growth) of the active material and the light weight of carbon fiber paper substrate. Such values were much higher than those (12–200 F g^−1^) of supercapacitors using metals as substrates.

## Results and Discussions

The direct growth of metal oxide/hydroxide on carbon materials is known to be difficult^17^. From the SEM images in some published papers, the amount of active material grown on the carbon fiber paper is generally too small^25^,^26^. Though nickel cobalt oxide has been reported to have good growth on certain carbon fiber papers^27^, unfortunately, we somehow failed to accomplish satisfying growth on untreated normal carbon fiber papers (CFPs) that were purchased commercially. To study the growth of active material on CFPs, self-hydrolysis of metal acetate precursors (i.e. nickel acetate and copper acetate) in water-ethanol mixtures was employed in this work. Any structure-directing templates, bases, agents, surfactants or post-treatments were avoided to achieve economy and efficiency. We anticipate that such an approach can not only simplify the electrode-preparation process, but also retain the purity of the resultant hydroxides. Another consideration of greater importance is that the polarity of ethanol is smaller than that of water, and so it may somewhat alleviate the hydrophobicity difference between the active materials and carbon fiber papers.

To decipher the effect of ethanol on the formation of the nanostructures, parallel experiments have been carried out. Detailed characterizations of the materials can be found in the Supporting Information. The crystal phase is copper incorporated α-nickel hydroxide (JCPDS Card No: 38-0715, referred to as nickel copper hydroxide NCH in the following texts) based on the SAED patterns (Figure S1). The elemental mapping results can be found in Figure S2. The surface chemical composition evolution can be determined by XPS, which is illustrated in Figure S3. The ratio of nickel to copper was about 0.8 to 0.2 according to ICP result. To simplify, we denote the nickel copper hydroxide grown on CFP with the volume concentration of ethanol at 0%, 30%, 50%, 70% and 100% as NCH-a, NCH-b, NCH-c, NCH-d and NCH-e in the following texts.

**Figure 1:**
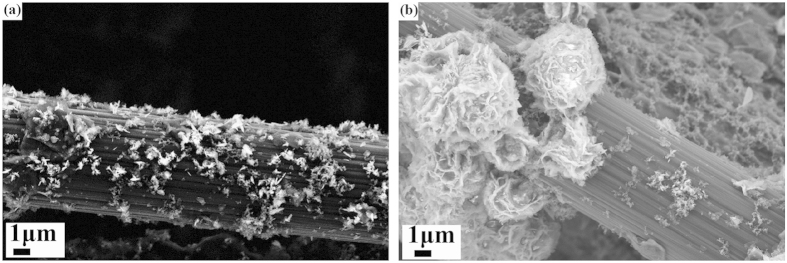
SEM images of two samples of NCH-a grown on carbon fiber papers (condition: pure deionized water as solvent).

The morphology of NCH when pure deionized water was used as the solvent is shown in Fig. 1. Samples prepared in separate runs were found to have the same limitations. From the images of two different NCH-a samples, the density of nano-clusters (NCH-a, 0% ethanol) was not high enough. Thus the whole carbon fiber surface could not be fully covered. Some parts of the surface of the bare fiber were still smooth and clean, identical to that of as-purchased carbon fiber paper. In addition, Fig. 1a,b show that the deposition of active material inside the framework of the CFP is non-uniform. It is found that the nanoflakes with different sizes were simply piled up on each other.

**Figure 2:**
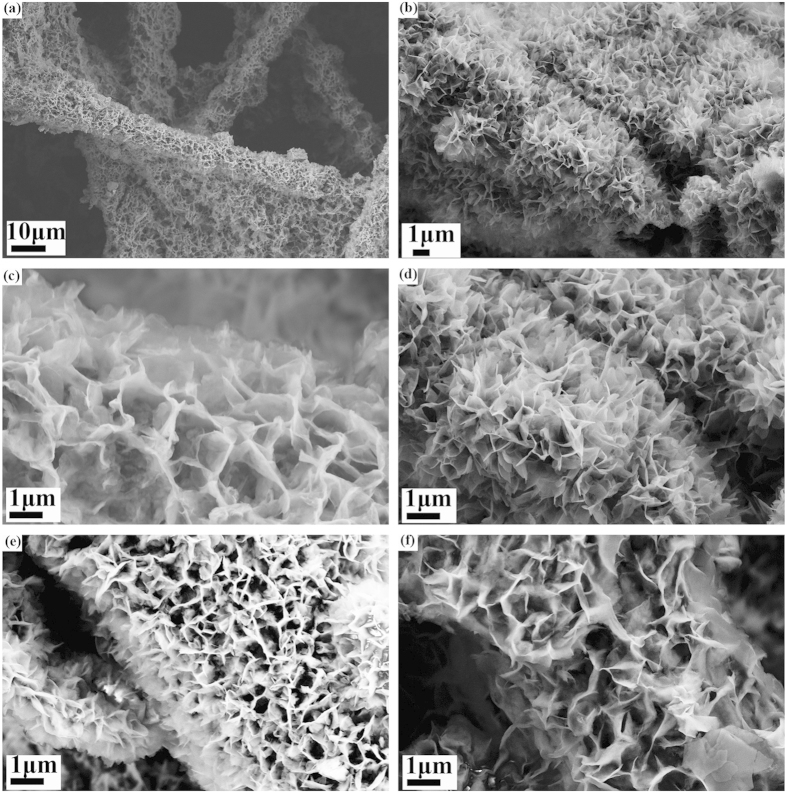
SEM images of nickel copper hydroxide (NCH) grown on carbon fiber paper (**a,b**) low magnification images of samples NCH-c to show the effect of ethanol on nickel hydroxide coverage on carbon paper; (**c**) sample NCH-b (30% ethanol); (**d**) sample NCH-c (50% ethanol); (**e**) sample NCH-d (70% ethanol); (**f**) sample NCH-e (100% ethanol).

In contrast, the morphologies of the material grown on CFP using water-ethanol mixtures as solvents were markedly improved. The SEM images of all the samples are shown in Fig. 2. Figure 2a,b display typical low magnification images of NCH-c (50% ethanol) grown on CFP. It can be seen that the material grown on CFP does not show bunching and aggregation. It is observed that the interlaced material thin flakes were uniformly coated over almost the entire skeletons of the carbon fiber paper; most of the carbon fibers were evenly wrapped by the material. The unique feature of material grown in the presence of ethanol is that each nanoflake has its own electrical/mechanical contact with the current collector. Thus, it is expected that the CFPs provide an effective electrical conduction path through the graphite fibers and good access to the electrolyte solution through the entire thickness of the substrate due to its three-dimensional microporous structure. Besides, the network configuration of nickel copper hydroxide could generate more active surface area and enhance the stability. When the volume of ethanol was high (NCH-d and NCH-e), the nanoflakes became big and some particles of the same material may coexist with the nanoflakes. Since the morphology of material grown in the presence of ethanol were quite similar, typical TEM images of the material grown in water (NCH-a) and in ethanol (NCH-c) have been examined. The results obtained, as shown in Figure S4, were consistent with the SEM images. The results demonstrated that ethanol was beneficial for the growth of NCH on the CFP, and the effect of its concentration on the morphology was not so pronounced in the mixed solvents. However, too much ethanol was detrimental.

To estimate the adhesion between NCH and CFP, we used ultrasonication to test whether the deposited active materials can be detached easily. After ultrasonication (37  kHz, 100 W) in pure water for half an hour, no visible particles were observed in the beaker containing sample NCH-c (50% ethanol). In contrast, floating particles were observed in the solution for the other beaker containing sample NCH-a (0% ethanol). The weight decrease for dried NCH-c was 3%, and that of dried NCH-a was 9%. From the results, together with the SEM images, it can be concluded that with the employment of ethanol, active materials can grow more firmly on the carbon fiber paper. In other words, the contact between the material and the substrate for the sample prepared employing ethanol is more intimate than that of the sample prepared in pure deionized water.

The formation mechanisms of copper incorporated α-nickel hydroxide are proposed as follows. It is reported^28^ that OH^-^ ions can be provided by three ways: (1) the hydrolysis of acetate (Equation 1); (2) the hydrolysis of ethanol (Equation 2); and (3) the cleavage of C-O bond in the ethanol derivative such as Ni_x_Cu_y_O(OH)_m_(OCH_2_CH_3_)_n_ (m+n = 1) in pure ethanol^29^:


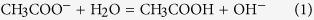



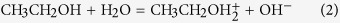


The gradual release of OH^−^ from hydrolysis of the acetate and ethanol at elevated temperatures can slow down the reaction rate of Ni^2+^ and Cu^2+^ with OH^−^30. The slowed-down reaction, on the other hand, can provide controlled nuclei formation and subsequent crystal growth (Equation 3). In addition, a slow production of OH^−^ that keeps the system buffered at near neutral pH can be considered as a key determinant, which is crucial for the synthesis of high-quality crystals.


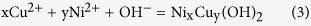


**Figure 3:**
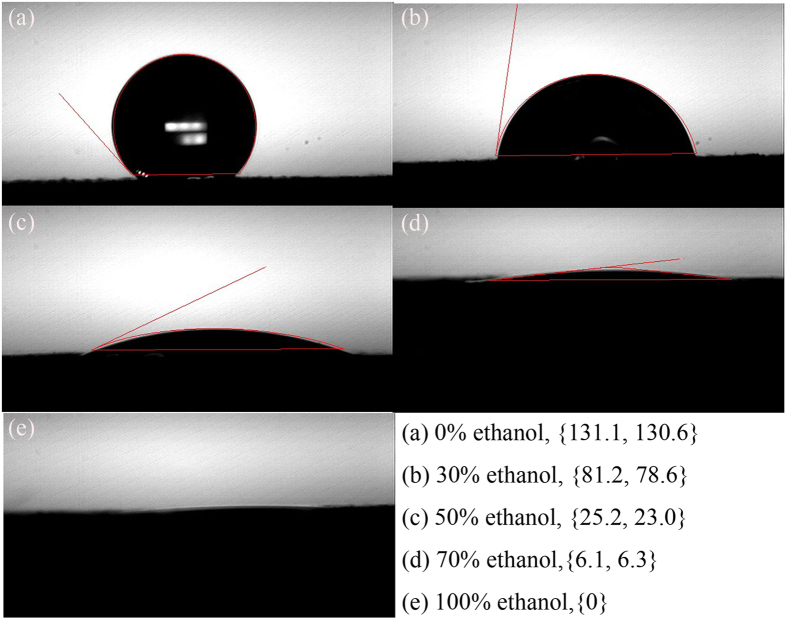
Contact angles between different solvents and carbon fiber paper. (**a**) 0% ethanol; (**b**) 30% ethanol; (**c**) 50% ethanol; (**d**) 70% ethanol; (**e**) 100% ethanol.

The aforementioned results demonstrate that nickel copper hydroxide can grow well on carbon fiber paper in the presence of ethanol. These results suggest that a high coverage or good growth of active material on carbon fiber does not necessarily require the presence of surface defects as commonly believed^18^. Amazingly, our results show that adding ethanol in the solution can also achieve superb coverage of active material on carbon. The reasons behind this phenomenon need exploration. We have searched literature, but cannot find the effect of ethanol on the growth of metal hydroxide on the carbon fiber paper. We believe that because carbon fiber is hydrophobic, it is hard for the aquatic solution to wet the surface of carbon fiber, leading to poor growth. To prove this, we performed contact angle measurements using different solvents (pure water, pure ethanol and their mixtures) on carbon fiber paper. The results can be found in Fig. 3. It is clear that with the addition of ethanol, the contact angle decreased significantly. This will make the nucleation of active material on carbon paper much easier in the solvent containing ethanol compared to that in pure DI water. The improved wettability is very important for the uniform hydrothermal growth in the subsequent steps, as revealed by the SEM images. This is also supported by the increase of loading of active material listed in Table 1.

**Figure 4:**
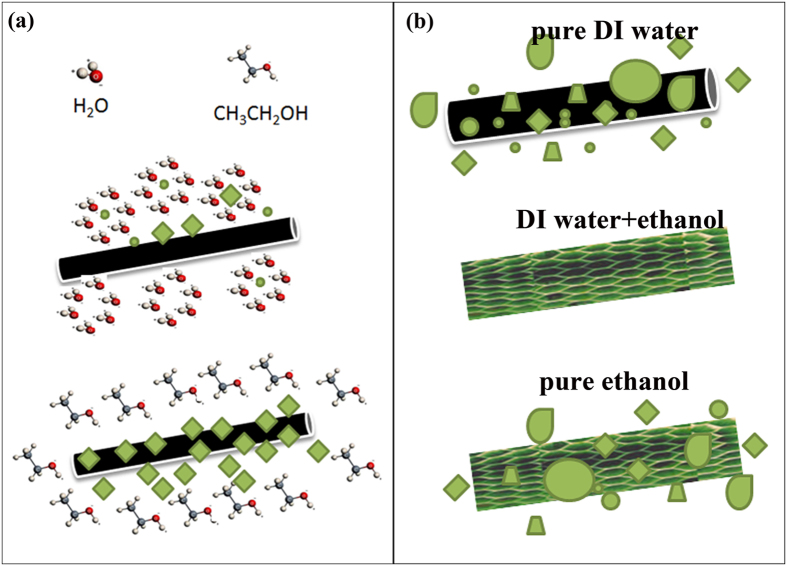
Schematic illustrations of the formation and growth of nickel copper hydroxide (NCH, green color in the Figure) on carbon fiber paper in different solvents. (**a**) Formation and attachment of NCH seeds on carbon fiber paper; (**b**) Final morphology of NCH in different solvents.

There is a variety of external factors that can have a strong impact on the solvothermal growth of crystals^31^. According to literature and considering our case, since precursors and reaction conditions (temperature and pressure) were kept the same, three factors (i.e. polarities, saturated vapor pressures and supersaturation) will affect the morphology and growth of samples synthesized^32^. Polarity can be seen most easily from the dielectric constants of the solvents, with a higher dielectric constant corresponding to a higher polarity. The dielectric constants of water and ethanol are 80.1 and 25.3 respectively^33^. The corresponding dipole moments of water and ethanol are 1.85 and 1.69 respectively^33^. Saturated vapor pressure is inversely related to the boiling point of the solvent. The boiling points of water and ethanol are 100.0 and 78.3 respectively^34^. The saturated vapor pressure of the solvents follows the reverse order. In a solution system, a low supersaturation results in inhomogeneous nucleation, which results in less uniformity of final products^35^. With the increase in ethanol, two contrary parameters will play important roles. The low dielectric constant and the low polarity of ethanol will increase the nucleation rate^36^, while the lower solubility of the precursors we use (nickel acetate and copper acetate) in ethanol than in water leads to a slower release of OH^−^37, which decrease the nucleation rate. Specifically, the carbon fiber paper we use is almost non-wettable in water. Thus, when the concentration of ethanol is zero, the nucleus is surrounded by water molecules and the CFP repels the water molecule and NCH nucleus, which make the direct nucleation and growth of NCH on CFP more difficult. When ethanol is introduced, more seeding sites of the nanostructures can be formed on the carbon fiber paper. Thus the materials are assembled in a way, generating much more nanoflakes grown on the carbon fiber paper than in pure DI water. The flakes mainly reside on the carbon fiber paper instead of at the periphery. When the percentage of ethanol is too high, the nucleation rate is also very high, thus some particles coexist with the nanoflakes. Schematic representation of the above discussion is clarified in Fig. 4. Figure 4a illustrates that the water molecule has higher polarity, and thus it can encircle hydrophilic NCH nucleus with surface hydroxyl group^38^,^39^, while ethanol will lessen such effect and favor the contact between the NCH nucleus and CFP. Figure 4b describes the subsequent growth of NCH with different ethanol concentrations. These explanations can also be supported by the SEM images.

**Figure 5:**
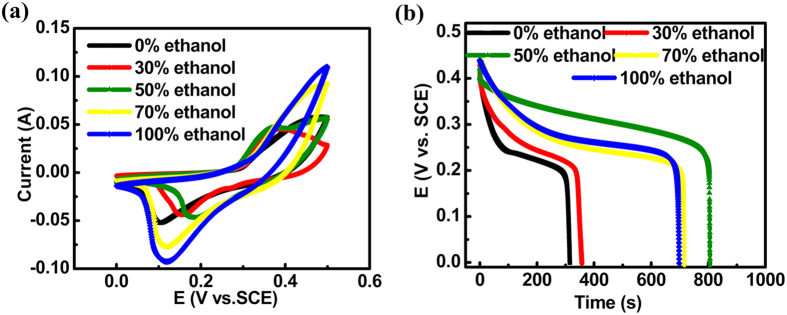
Comparison of (**a**) CV curves and (**b**) discharge curves of different samples synthesized at different solvents.

To investigate the effect of ethanol on the electrochemical properties, the as-prepared composite samples (active materials and carbon fiber papers) were evaluated in 1 mol L^−1^ KOH electrolyte. The electrochemical properties of all the samples obtained at different concentrations of ethanol are compared in Fig. 5. When pure deionized water (NCH-a) or pure ethanol (NCH-e) was used as the solvent, the separation of redox peak in the CV curves was bigger, implying that the electrochemical reversibility was not good if only water or ethanol was used. The specific capacitances calculated from the discharge curves for NCH-a, NCH-b, NCH-c, NCH-d and NCH-e are listed in Table 1. Galvanostatic charge-discharge (GCD) results are used for comparison because this measurement is reported to be the most reliable and accurate method for evaluating the capacitance of electrodes compared to either cyclic voltammetry or impedimetric methods^40^,^41^,^42^. Corresponding specific capacitances per total weight of the whole electrodes (CFP plus NCH) are also listed. The trend of the change was reasonable and consistent with the SEM results. When the volume concentration of ethanol was 50%, the flakes were thin and the coverage was excellent without any particles or aggregations, which endowed fast transportation and more accessible sites for ions. A comparison of the electrochemical performance of various metal oxide/hydroxide on carbon fiber paper based supercapacitors published recently in literature is displayed in Table 2. Attractively, the capacitance of our result per total weight of the active material and the substrate was 596 F g^−1^, much higher than the rest, even higher than RuO_2_ grown on CFP (84 F g^−1^), which showed the competitive advantage of our approach.

**Figure 6:**
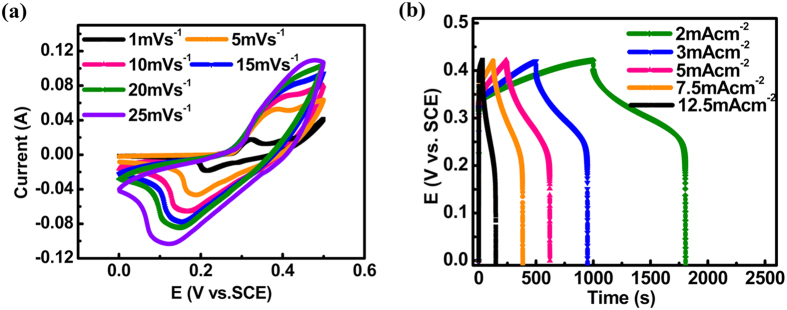
(**a**) CV curves at different scan rates (**b**) GCD curves at different discharge currents of the electrodes synthesized in mixed solvent of water and ethanol (NCH-c, percentage of ethanol:50%) for supercapacitors application.

NCH-c (50% ethanol) is the best sample among all the NCH/CFP composites as stated in Table 1, thus detailed discussion is added here. The cyclic voltammetry (CV) curves of the NCH-c at scan rates ranging from 1 to 25 mV s^−1^ are shown in Fig. 6a. The redox current peaks during the anodic and cathodic sweeps were explicitly observed for each electrode, which can be attributed to the intercalation/extraction of OH^−^ ions into/out of nickel copper hydroxide interlayer’s and the simultaneous reduction/oxidation of Ni^2+^ ions. It is interesting that one couple instead of two couples of redox peaks appeared. One explanation is that the redox peak of copper overlaps with nickel and becomes a broad peak; the other is that copper does not contribute to the electrochemical performance, and it only serves as an element to stabilize α-Ni(OH)_2_. It is exhibited in the Supporting Information Figure S5 that carbon fiber paper itself had negligible current response compared to the one with active materials. This potential contribution from the carbon fiber paper itself when calculating specific capacitance can be ruled out. Galvanostatic charge-discharge profiles are shown in Fig. 6b. The specific capacitance calculated based on the weight of active material from charge-discharge can reach up to 1397, 1362, 1798, 1766, 1258 F g^−1^ at current densities of 2, 3, 5, 7.5, 12.5 mA cm^-2^. The lower specific capacitances at 2 and 3 mA cm^−2^ can be because the test was started from the smallest current upwards. Thus, the charge storing ability of the material was not fully activated initially. The results showed that the capacitance measured at 12.5 mA cm^−2^ is degraded only by 10% compared to the capacitance measured at 2 mA cm^−2^, which suggested good rate capability. No obvious decrease in capacitance was observed for the supercapacitor at increasing current densities, showing that the supercapacitor can effectively work at a wide range of current densities. The uniform distribution without cracks, ample utilized ion-accessible surface area and intimate contact are the most likely reasons for the significant improvement in rate capability. The specific surface areas of all the samples were found to be 47, 35, 58, 26 and 13 m^2^ g^−1^ as calculated by the Brunauer-Emmett-Teller (BET) method. We notice that the capacitance at higher current density can even be larger than that at lower current density. The calculated capacitances per total weight of carbon fiber paper plus active material achieved 598, 584,770,757,539 F g^−1^ at current densities of 2, 3, 5, 7.5, 12.5 mA cm^−2^.

**Figure 7:**
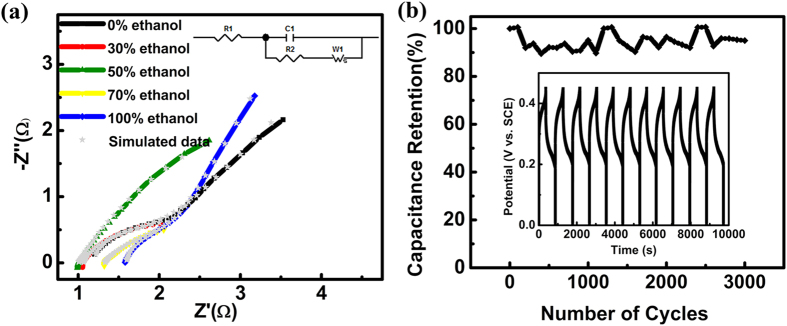
(**a**) Electrochemical impedance spectra of NCH/CFP hierarchical structures; (**b**) Capacitance retention vs. cycle number of the nickel copper hydroxide material.

The cycling stability of NCH-c was evaluated by repeating the galvanostatic charge-discharge tests at a current density of 5 mA cm^−2^ for 3000 cycles, as depicted in Fig. 7b. No remarkable capacitance decay appeared during the 3000 cycles. The capacitance went through a small variation (10% at most) during the cycling. These results demonstrated that the as-prepared sample was very stable during the cycling test. The solution remained transparent after the cycling, which indicated a minimal dissolution of nickel copper hydroxide into the solution, suggesting that the bottom of NCH is anchored to the surface of the substrate.

To investigate the effect of ethanol on the electrode kinetics, electrochemical impedance spectroscopy (EIS) measurements were performed for all samples, as illustrated in Fig. 7a. Both the sample prepared in pure deionized water and the samples prepared in the mixture of ethanol and water demonstrated a similar form with an arc in the higher frequency region and a spike at a lower frequency. At high frequency, the intercept of the curve at the real part indicates the resistance of the electrochemical system (*R*_ESR_, which includes the inherent resistance of the electroactive material, ionic resistance of electrolyte, and contact resistance at the interface between electrolyte and electrode)^43^,^44^. The corresponding *R*_ESR_ value was very low, being 1.18, 1.06, 1.00, 1.32 and 1.60 Ω for the 0%, 30%, 50%, 70% and 100% volume concentration of ethanol (from NCH-a to NCH-e), respectively from the fitting data. The sample with the lowest *R*_ESR_ value was found having the highest capacitance as showed in Table 1, suggesting the importance of electron transport. The different trends of *R*_ESR_ and capacitance values can be due to the weight difference of the active materials shown in Table 1. At the low frequency region, the linear part of the impedance plots is corresponding to Warburg impedance W, which is described as a diffusive resistance of OH^−^ inside NCH/CFP electrode. We used an equivalent circuit to obtain the Warburg constants of the five samples. The results were 2.9, 20.9, 6.7, 22.1 and 2.9 Ω s^−1/2^, for sample prepared using 0%, 30%, 50%, 70% and 100% ethanol concentrations, respectively. The Warburg slopes for samples synthesized using 0%, 50% and 100% ethanol were higher than for the other samples, indicating faster ion diffusion for their ordered intercalation structure and more uniform pore size distribution. Correlation of the capacitance and Warburg constant appeared to be present for samples prepared with 30%, 50% and 70% of ethanol concentration, but we could not understand why they were not present for the other two samples.

**Figure 8:**
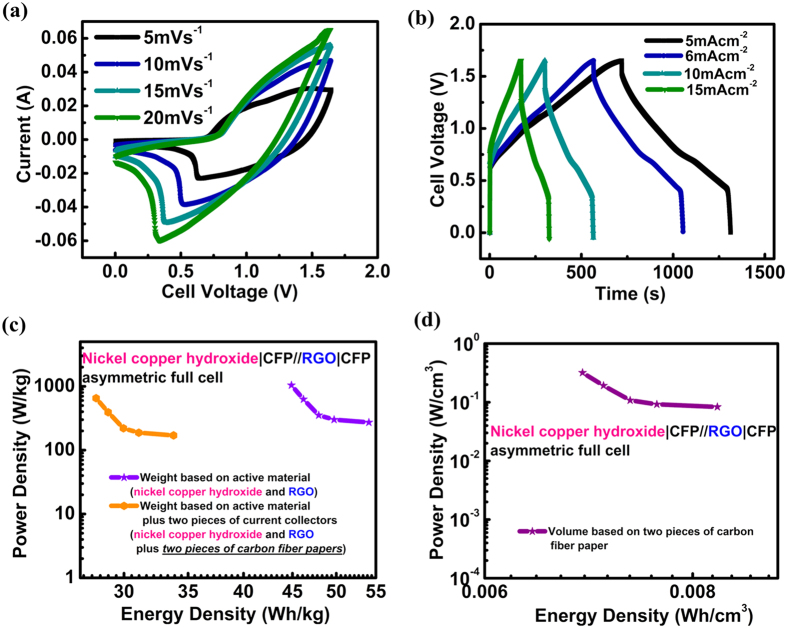
(**a**) CV curves of the full cell at various scan rates; (**b**) Charge–discharge curves of the cell at various constant current; (**c**) Ragone plots of the asymmetric supercapacitor on a weight base; (**d**) Ragone plots of the asymmetric supercapacitor on a volume base.

On account of the good performance of NCH-c/CFP prepared with the assistance of ethanol, and to further evaluate the practical application potential, an asymmetric supercapacitor (ASC) has been made by using the NCH-c/CFP electrode as the cathode (since NCH-c has shown the best performance) and the reduced graphene oxide (RGO) on another CFP as the anode. For supercapacitors, it is well-known that the charge balance between the positive and negative electrodes should follow the relationship *q*^+^ = *q*^−^, where the stored charges are related to the specific capacitance (*C*), the potential window (*U*), and the mass of the electrode (m) according to *q* = *CUm*^45^. The potential window of RGO is 1 V while that of NCH-c is 0.5 V. The capacitance of RGO is around 140 F g^−1^, while that of NCH-c is around 1400 F g^−1^. Therefore, the optimal mass ratio between the electrodes should be around 5 in the present ASC device. The loading amount of positive and negative active materials is 5 mg and 25 mg, respectively, with the total loading of 30 mg. Figure 8a exhibits CV curves of the electrode-optimized supercapacitor device at different scan rates from 0 to 1.65 V. It displayed a quasi-rectangular CV geometry as EDLC, indicating the fast charge propagation within the electrodes. Galvanostatic charge-discharge curves at various current densities are further illustrated in Fig. 8b. The specific capacitance can reach 143 F g^−1^ at 1 mA cm^−2^ and still maintains 118 F g^−1^ at 15 mA cm^−2^. Figure 8c,d shows the Ragone plot (i.e., energy density vs. power density) of ASC device based on weight and volume of the positive and negative electrodes. The NCH//CFP asymmetric cell exhibited an energy density of 54 Wh kg^−1^ per total weight of active materials of both electrodes (nickel copper hydroxide plus reduced graphene oxide) at a power density of 272 W kg^−1^, and maintained 44 Wh kg^−1^ at a power density of 1042 W kg^−1^. Extraordinarily, the energy density value per total weight of both electrodes and two pieces of carbon paper substrates in the device can reach 33 Wh kg^−1^ at a power density of 170 W kg^−1^. And maintained at a value of 28 Wh kg^−1^ at a power density of 651 W kg^-1^. To show the superiority, we use our other similar sample (nickel copper oxide) grown on nickel foam as comparison^46^. The energy density can reach 90 Wh kg^−1^ per total weight of the electrodes. However, when the weights of nickel foams are included, the energy density decreases to one-ninth, reaching a value of 10 Wh kg^−1^. It is impressive that when the weight of two pieces of carbon fiber paper is added, the value only decreases by half, indicating the great advantage in using carbon paper as current collector or substrate, as the total weight of the device is much lower than devices using metal foams. Not so many papers nowadays report the energy density and power density based on the total weight of the positive and negative electrodes (together with the current collector). Thus it is very hard to compare the energy density per total weight of electrodes and substrate with theirs. However, we note that the energy density per total weight of electrodes and substrate of our NCH-c/CFP device is comparable to the energy density of other high performance devices in the literature, even if only the weight of the active material is considered in their reports (46.7 Wh kg^−1^ for MnO_2_//GO and 41.2 Wh kg^−1^ for MoO_3_//C)^47^,^48^. Moreover, some papers adopt the vacuum filtration method and avoid the use of current collector when they use graphene as both the support and conductive path for nickel hydroxide^31^. The highest energy density they achieved is 18 Wh kg^−1^. Our advantage is that the carbon fiber paper is cheaper than graphene, which can be commercialized in the future. Also, the total energy of our devices is very high (48 mg × 33 Wh kg^−1^ = 1.58 mWh) when compared with those with limited active materials loading.

The good electrochemical performance of the NCH//CFP can be mainly attributed to the following factors: firstly, the ultrathin nanoflakes interconnected with each other constitute well-arranged networks on the surface of carbon fiber prepared in the presence of ethanol, resulting in a large contact area and integrated electrode configuration; secondly, the loose and open channels within the electrode matrix provide efficient and fast electron and ion transport pathways; and thirdly, the electrode material prepared using only water and ethanol in addition to metal salts is of high purity and allows fast charge-transfer.

## Conclusion

In summary, nickel copper hydroxide could successfully grow on carbon fiber paper with the employment of ethanol in the synthesis solution by a one-step facile method using only acetates as the precursors. The effect of ethanol on the growth and electrochemical performance has been investigated. It is found that ethanol can greatly enhance the growth of metal hydroxide material on the carbon fiber paper. The low-cost and sustainable preparation method to grow active material with high quality on lightweight current collectors has significant potential for wearable and lightweight energy storage devices. These architectures with good contact show superior electrochemical storage properties even based on the total weight of the electrodes as a whole (the weight of carbon fiber paper is also included). The capacitance from three-electrode configuration can reach 770 F g^−1^ per total weight of both active material and carbon fiber paper substrate. The two-electrode asymmetric supercapacitor could lead to high gravimetric, areal and volumetric energy densities of 33 Wh kg^−1^, 0.4 mWh cm^−2^ and 8 mWh cm^−3^, respectively. More importantly, this fabrication of high performance full cell without metal current collectors and binders signifies the possibility of achieving a supercapacitor with very high capacitance and greatly reduced weight.

## Experimental Section

### Synthesis of nickel copper hydroxide on carbon fiber paper

In a typical synthesis, nickel acetate (2 mmol) and copper acetate (0.5 mmol) were dissolved in 25 mL of deionized water. After the solution was stirred for 30 min, the above solution was transferred into a Teflon-lined stainless steel autoclave. A piece of carbon fiber paper (bought from Full Cell Earth 2 × 2 cm, about 0.012 g, after washing in acetone, ethanol and deionized water) has been put into the autoclave. Hydrothermal synthesis was run at 120 °C for 8 h and then naturally cooled down to room temperature. Afterwards, the substrates were taken out and cleaned by ultrasonication for several minutes to remove the loosely attached products on the surface, and then dried at 80 °C in an oven overnight. A series of experiments were designed to study the influences of the ratio of water to ethanol in volume on the growth of nickel copper hydroxide (NCH) on the carbon fiber paper.

### Characterizations

The morphology of the prepared samples was examined by using a scanning electron microscope with an X-ray energy dispersive spectrometer (SEM, Zeiss SUPRA40). The crystal structure was identified using X-ray diffraction. Transmission electron microscopy and electron diffraction (TEM, JEOL 2000FX) were employed to obtain SAED patterns. The composition of the products was examined by energy-dispersive X-ray spectroscopy (EDX) and ion chromatography (IC) analysis - modular IC system. The surface functional groups were investigated by X-ray photoelectron spectroscopy (XPS) (AXIS Ultra). Electrochemical measurements were carried out in aqueous KOH (1 M) where NCH was directly used as the working electrode without any polymer binder or conductive addictives, a platinum foil as the counter electrode, and a standard calomel electrode (SCE) as the reference electrode. Electrochemical impedance spectroscopy (EIS) measurements were conducted by applying an AC voltage with 5 mV amplitude in a frequency range from 0.01 Hz to 100 kHz at the open circuit potential. All of the electrochemical measurements were performed on a Solartron 1470E multichannel potentiostat/cell test system.

### Fabrication of ASCs

The ASCs were assembled by using NCH as the positive electrode and reduced graphene oxide as the negative electrode. Briefly, the NCH/CFP (2 × 1 cm in a rectangular shape) directly acted as the positive electrode without any ancillary materials. RGO (Graphene Supermarket) and carbon black were mixed with a weight ratio of 80:20 and dispersed in ethanol to obtain a slurry, which was then coated onto the CFP current collector (2 × 2 cm) with a spatula. Finally, the as-prepared negative electrode was dried in an oven at 80 °C for 24 h. The mass loading of nickel copper hydroxide was around 2.5 mg cm^−2^, while the loading of RGO and carbon black was around 6.25 mg cm^−2^. The total weight of both positive electrode (nickel copper hydroxide (5 mg) plus carbon fiber paper (6 mg) is 0.011 g) and negative electrode (25 mg) plus carbon fiber paper (12 mg) is 0.037 g) is 0.048 g (48 mg).

## Additional Information

**How to cite this article**: Zhang, L. and Gong, H. A cheap and non-destructive approach to increase coverage/loading of hydrophilic hydroxide on hydrophobic carbon for lightweight and high-performance supercapacitors. *Sci. Rep.* **5**, 18108; doi: 10.1038/srep18108 (2015).

## Supplementary Material

Supplementary Information

## Figures and Tables

**Table 1:** Capacitances and active material loading of NCH/carbon fiber paper (CFP) based supercapacitors with respect to different solvents calculated based on galvanostatic charge-discharge profiles at the current densities of 2 mA cm^−2^.

	Volume concentration of ethanol	Active Material Loading (mg cm^−2^)	Areal Capacitance (F cm^−2^)	Specific Capacitance per weight of active material (Fg^−1^)	Specific Capacitance per total weight of active material and substrate (Fg^−1^)
NCH-a	0%	1.75	1.39	794	292
NCH-b	30%	2	1.59	783	319
NCH-c	50%	2.25	3.14	1362	598
NCH-d	70%	2.5	2.96	1134	539
NCH-e	100%	2.75	2.88	1049	501

**Table 2:** Electrochemical performance of metal oxide/hydroxide on carbon fiber paper (CFP) based supercapacitors.

Specimen Structure	Current Density/Scan Rate	Active Material loading (mg cm^−2^)	Areal Capacitance (F cm^−2^)	Specific Capacitance (Fg^−1^)	Specific Capacitance (The weight of cfp is added) (Fg^−1^)	Year Published
NiO/CFP	1 mA cm^−2^	1.1	0.93	840	84	2013^49^
Co_0.67_Ni_0.33_ DHs/NiCo_2_O_4_/CFP	2 mA cm^−2^	1	1.64	1500	—	2013^50^
RuO_2_/CNT film	20 mVs^−1^	0.128	0.149	1176	142 (calculated)	2005^51^
MnOx/CFP	0.61 A/g	0.16	0.64	2530	63	2012^27^
MnO_2_/Graphene	2 mVs^-1^	9.8	1.42	465	130	2013^52^
NCH/CFP	2 mAcm^−2^	2.25	3.14	1362	598	Current work

(The values reported for the other supercapacitor devices are added for comparison).
