# Supplementary material for: A cheap and non-destructive approach to increase coverage/loading of hydrophilic hydroxide on hydrophobic carbon for lightweight and high-performance supercapacitors
Source: Sci Rep. 2015 Dec 8;5:18108. doi: 10.1038/srep18108 (PMC4672290; doi:10.1038/srep18108)
Supplement: Supplementary Information [file srep18108-s1.pdf]

A cheap and non-destructive approach to increase coverage/loading of hydrophilic hydroxide on hydrophobic carbon for lightweight and high-performance supercapacitors

Liuyang Zhang, Hao Gong*

Department of Material Science and Engineering, National University of Singapore, Singapore 117576, Singapore

Correspondence and requests for materials should be addressed to H. Gong (**msegongh@nus.edu.sg**)

Characterization of the material synthesized (Nickel copper hydroxide: NCH)

To examine the crystal structure of the material, X-ray diffraction has been used. Unfortunately, carbon fiber paper itself has so many peaks and there is little difference between the material grown on it and the pure carbon fiber paper. Thus, we resort to TEM diffraction to scrutinize. The SAED patterns of all the materials have been exhibited in **Figure S1**. The calculated lattice spacings are quite similar and the results have been listed in Figure 2a, which means that the concentration of ethanol did not influence the phase of the final products. Except for the second ring, all the others can be indexed to α-nickel hydroxide (JCPDS Card No: 38-0715) with an expansion of lattice constant at about 1.02. It is noteworthy that the less stable phase α-$Ni(OH)_2$ instead of the more stable phase β-$Ni(OH)_2$ is synthesized , which indicates that the incorporation of copper can restrict the transformation and stabilize the α-$Ni(OH)_2$, which is favorable since α-phase could deliver a higher

capacitance than β-phase. The second ring may be due to the intercalation of some anions into the (009) layer of nickel hydroxide. It is interesting that the copper hydroxide is not formed. There is no inhomogeneity in the morphology as well as in crystal structures. A uniform distribution of metal cations on the atomic level without segregation is achieved.

Based on our previous studies, the ratio of nickel to copper has been optimized to be 4 to 1 in the precursors to achieve the highest supercapacitor performance. To determine the ratio of nickel to copper and distribution in the final product, ion chromatography (IC) analysis and energy-dispersive X-ray spectra mapping have been conducted. **Figure S2** elucidates the uniform distribution of nickel, copper and oxygen. The IC analysis result reveals the ratio of nickel to copper is 699.3 to 181.4, which is quite close to that of 4:1 in the precursors.

To assess the chemical composition evolution of the nickel copper hydroxide, X-ray photoelectron spectroscopy (XPS) were measured. The XPS survey spectra show that there is only nickel, copper and oxygen existing on the surface, revealing the impurity level of our material is low. The surface information of the NCO/Cu could be found from the XPS results displayed in **Figure S3**. The peaks and their corresponding satellites at 853 and 871 eV (Figure 4a) can be interpreted as a nickel (II) state according to literature[1]. This is also the case for Cu 2p XPS spectra, and the binding energy of 932.5 eV and 952.1 eV (Figure 4b) can be attributed to Cu^{2+} $2p_{3/2}$ and $2p_{1/2}$[2].

Calculations

The mass and area specific capacitances can be calculated from the CV curve using the following equations[3,4]

$$C = \frac{\int Ivdv}{2vmU} \qquad (1)$$

$$C = \frac{\int Ivdv}{2vAU} \quad (2)$$

where I (A) and U (V) are the current and potential in the CV, v (V s^{-1}) is the scan rate, A (cm^2) is the area of the current collector together with the active material, U is the potential window of discharge (0.5 V here), I is the constant discharge current and t (s) is the discharge time.

The mass and area specific capacitances, power density and energy density are calculated based on the galvanic charging-discharging curves using the equation as follows[5,6]:

$$C = \frac{It}{mU} \quad (3)$$

$$C = \frac{It}{AU} \quad (4)$$

$$E = \frac{1}{2} \times C \times U^2 \quad (5)$$

$$P = \frac{E}{t} \quad (6)$$

where C (F g^{-1}) is specific capacitance, A is the area of the current collector, E (Wh kg^{-1}) is energy density, P (W kg^{-1}) is power density, U is potential window (here 1.7 V), I (A) is discharge current, t (s) is discharge time, m (g) is the sum of the masses of the positive electrode (NCH, here 8 mg) and negative electrode(reduced graphene oxide, here 20 mg), thus here m is 28 mg.

The areal energy density could be estimated by

E_a=E_g×m / (2 cm×2 cm) (The area of two pieces of carbon fiber paper)

P_a=P_g×m / (2 cm×2 cm)

The volumetric energy density could be estimated by

E_v=E_g×m / (2 cm×2 cm×0.049 cm) (The volume of two pieces of carbon fiber paper)

$P_v = P_g \times m / (2\ \text{cm} \times 2\ \text{cm} \times 0.049\ \text{cm})$

All the above parameters are based on the weight of active material, we also calculate and report all the parameters based on the weight of total electrode, i.e. active material and the weight of carbon fiber paper.

In the three electrode configuration, the weight of carbon fiber paper which is 12 mg is added. In the two electrode configuration, the weight of two pieces of carbon fiber paper, which is 24 mg, is added**.**

(a) Ring number in SAED patterns	D value from SEAD pattern (Å)	D value of α-$Ni(OH)_2$ from PDF Card (Å)	(hkl) for α-$Ni(OH)_2$
1	3.97-4.03	3.90	(006)
2	3.15-3.24		
3	2.7-2.76	2.68	(101)
4	1.58-1.61	1.54	(110)
5	1.36-1.38	1.33	(119)
6	1.03-1.05	1.01	(210)
7	0.91-0.92	0.89	(310)

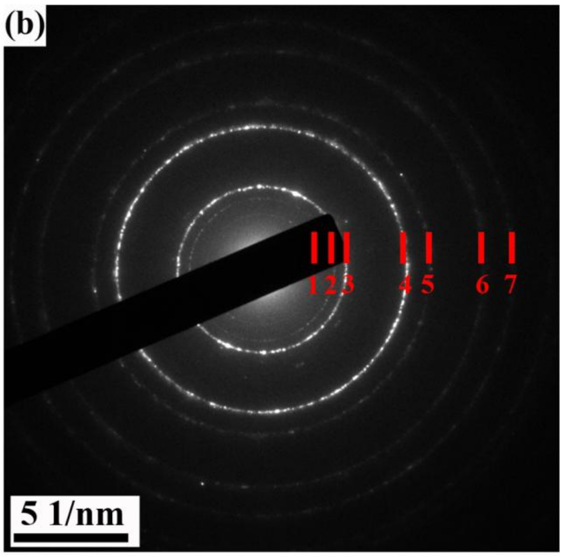

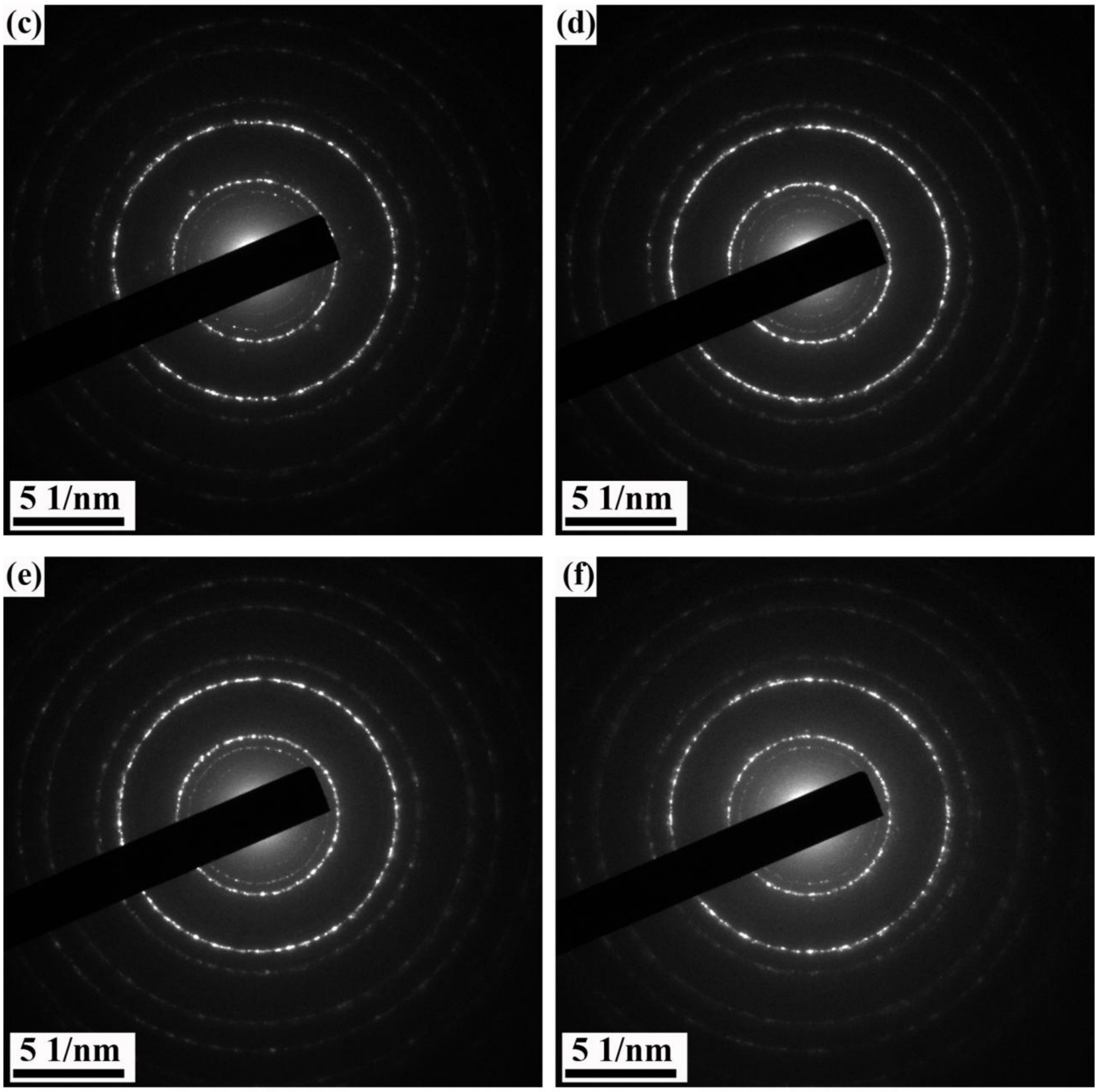

Figure S1. (a) Calculated D value from SAED patterns compared with JCPDS card; (b-f) Selected area electron diffraction (SAED) patterns of materials synthesized with different volume ratios of water to ethanol:(b) sample

NCH-b (0% ethanol); (c) sample NCH-c (30% ethanol); (d) sample NCH-d (50% ethanol); (e) sample NCH-e (70% ethanol); (f) sample NCH-f (100% ethanol).

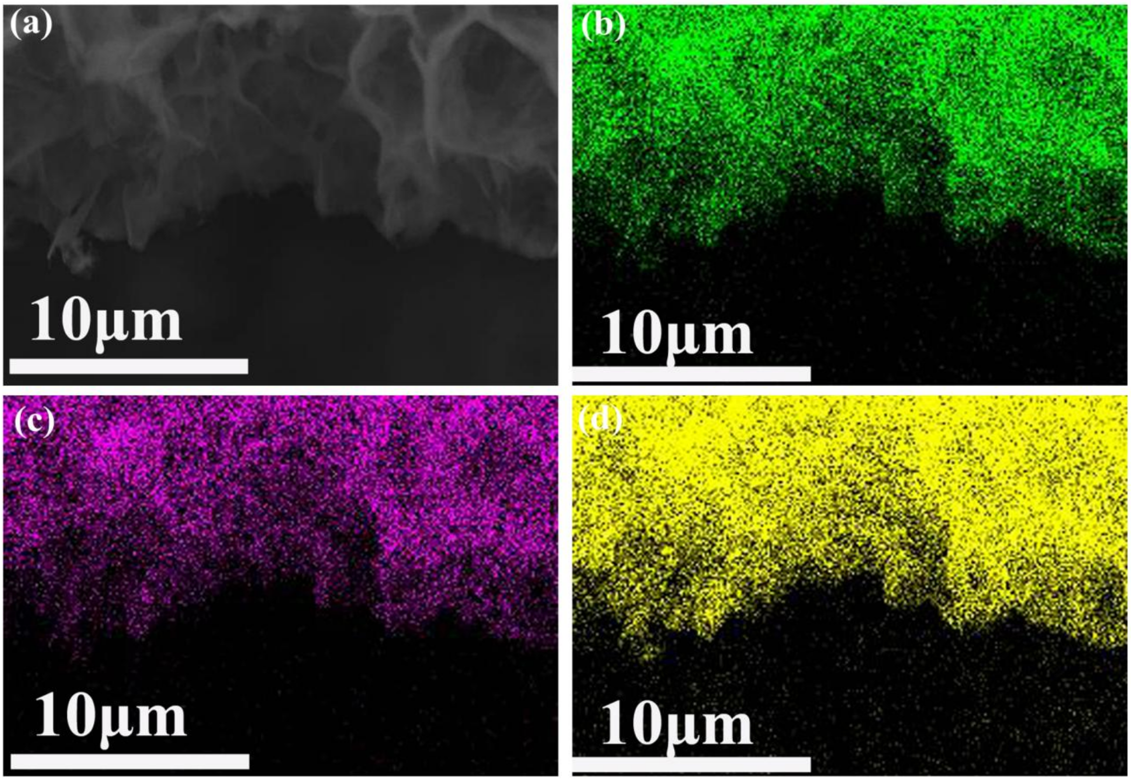

Figure S2. SEM Mapping of the material (a) image (b) Ni (c) Cu (d) O.

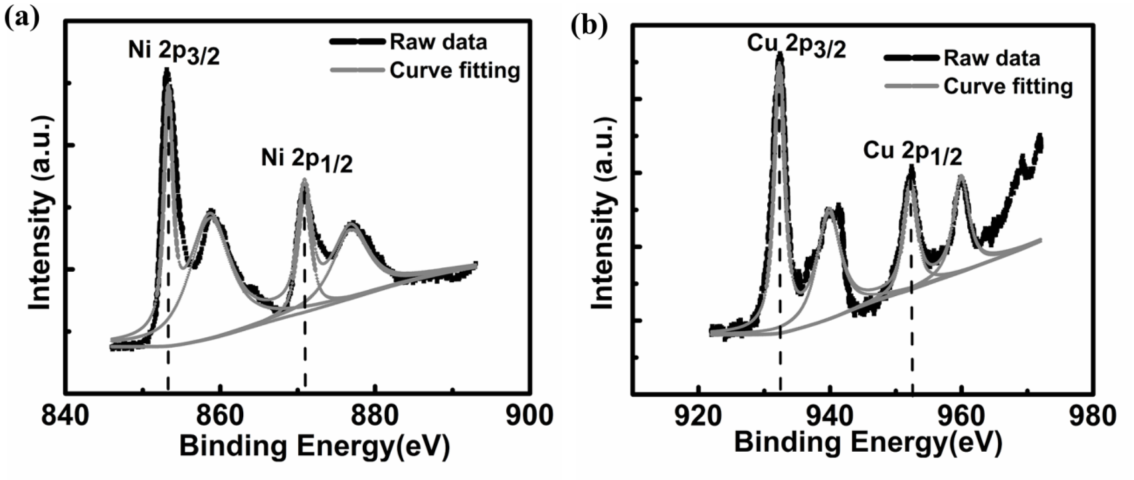

Figure S3. XPS high-resolution spectra and curve fitting data of nickel copper hydroxide (NCH-d) (a) Ni 2p; (b) Cu 2p.

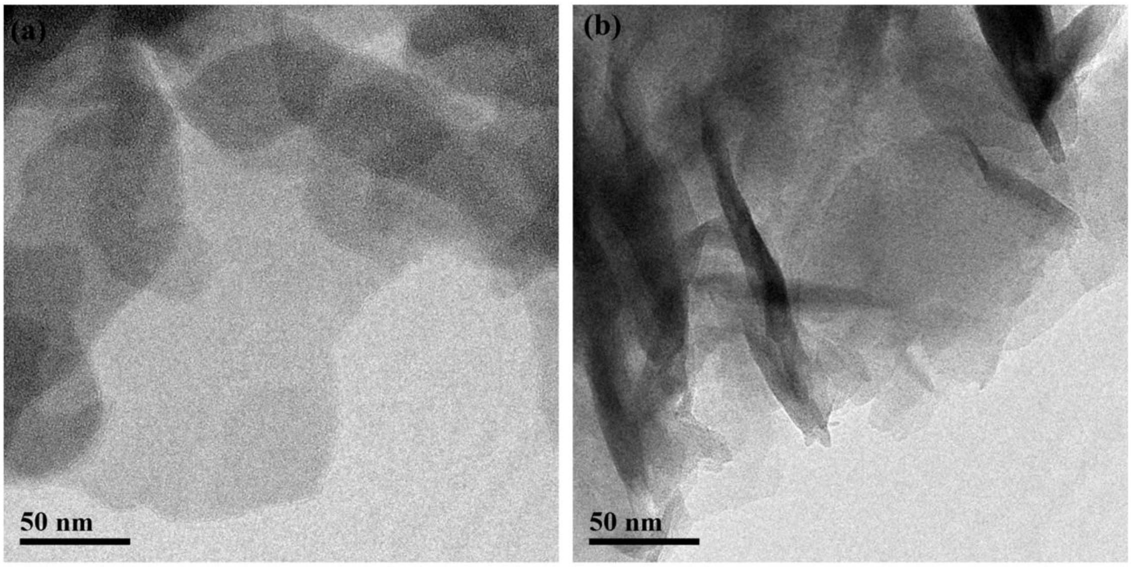

Figures S4. Typical TEM images of NCH synthesized with different solvents (a) pure deionized water (b) mixed solvents of ethanol and deionized water.

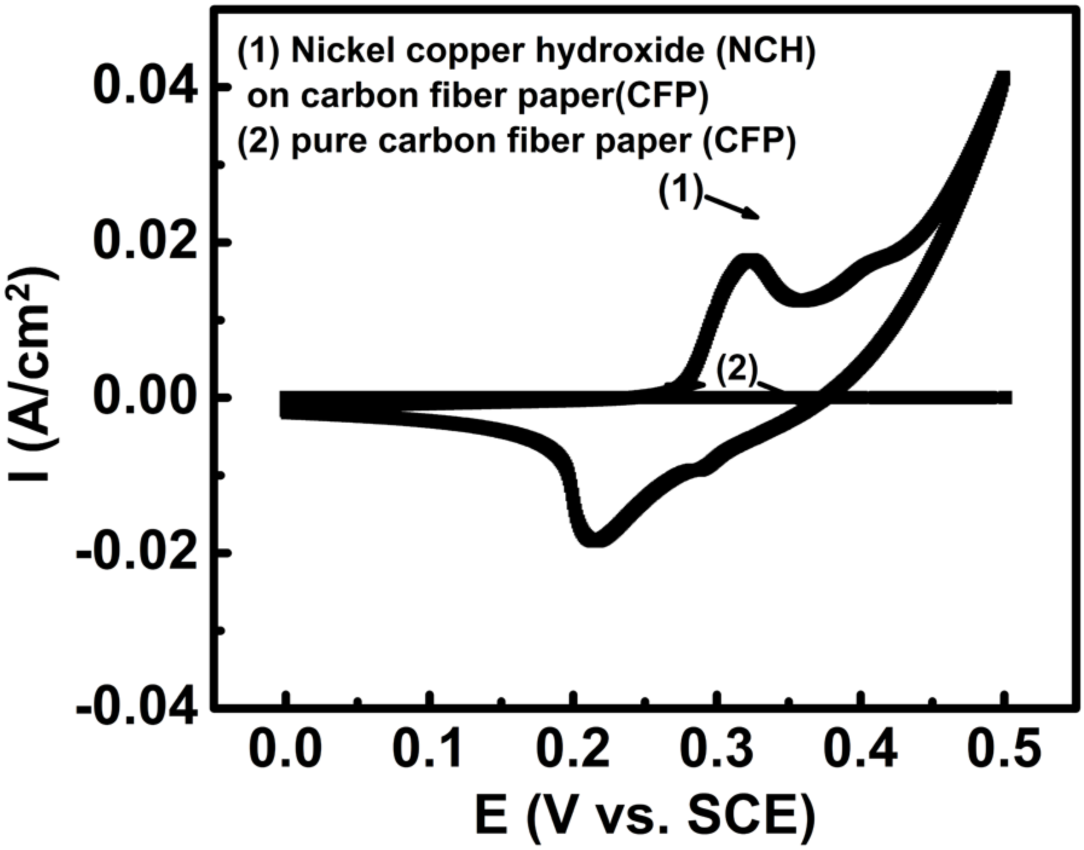

Figure S5. Comparison of CV curves at 1mV s^{-1} between NCH grown on carbon fiber paper and the pure carbon fiber paper.

References

1. Fominykh, K. *et al.* Ultrasmall dispersible crystalline nickel oxide nanoparticles as high-performance catalysts for electrochemical water splitting. *Adv. Funct. Mater.* **24,** 3123-3129 (2014).
2. Wu, C. K., Yin, M., O'Brien, S., Koberstein, J. T. Quantitative analysis of copper oxide nanoparticle composition and structure by X-ray photoelectron spectroscopy. *Chem. Mater.* **18,** 6054-6058 (2006).
3. Conway, B. E. *Electrochemical Supercapacitors: Scientific Fundamentals and Technological Applications* Ch. 15, 417-475 (Kluwer Academic / Plenum Publishers: New York, 1997)
4. Huang, L., Chen, D., Ding, Y.,Feng, S., Wang, Z. L., Liu,M. Nickel–cobalt hydroxide nanosheets coated on $NiCo_2O_4$ nanowires grown on carbon fiber paper for high-performance pseudocapacitors. *Nano Lett.* **13,** 3135-3139 (2013).
5. Stoller, M. D., Ruoff, R. S. Best practice methods for determining an electrode material's performance for ultracapacitors. *Energy Environ. Sci.* **3,** 1294-1301 (2010).
6. Hulicova-Jurcakova, D., Puziy, A. M., Poddubnaya, O. I., Suarez-Garcıa, F., Tascon, J. M. D., Lu, G. Q. Highly stable performance of supercapacitors from phosphorus-enriched carbons. *J. Am. Chem. Soc.* **131,** 5026-5027 (2009).
